# The War and Medical Teaching

**Published:** 1915-01-23

**Authors:** 


### The Hospital

The Workers' Newspaper of Administrative Medicine and Institutional Life, Administration, National Insurance and Health.

No. 1492, Vol. LVII. SATURDAY, JANUARY 23, 1915. PRICE ONE PENNY.

### THE WAR AND MEDICAL TEACHING.

To take occasion by the hand is an excellent policy for those on reformations bent. Suggestions and proposals which in ordinary circumstances are treated with cold neglect may hope for a very different welcome when the times are propitious. Hence the existence of a state of war has brought into the columns of our newspapers the inventor of some specially powerful explosive or other apparatus of destruction, and the holder of some secret which will establish the welfare of the nation were only a modest contribution of capital forthcoming. These gentlemen but a few months ago would have prophesied to a heedless public, but with Europe ablaze they see their opportunity. The current serves, and they hopefully launch their ventures.

It seems a far cry from a European war to the methods of medical teaching, but there are ingenious minds which see a connection between the two. The chance is too good to be lost. A public which has hitherto been deaf and careless may now perchance be aroused to attention, and voices previously lost in the wilderness may gain a ready audience. It is doubtless under convictions of this order that an attempt is made to discuss once more a modification of the medical curriculum in the direction of the apprenticeship system. The war promises a scanty supply of doctors; this would be less severe were a four years' hospital curriculum, with one year of apprenticeship, substituted for the present arrangement; therefore why not allow fifth-year students to act as unqualified assistants and so save the situation both now and in the future? This is the sum of the argument, and it is intended to show that a proposal virtuous even in times of peace is not unequal even to the existing emergency. It is impossible to abstain from admiring both the abiding faith of the reformer in his method and the ingenuity which sees in present circumstances an opportunity for its advocacy. The hour has come and the man is not wanting. All the old and well-worn controversies in reference to the unqualified assistant, the advantages and disadvantages of apprenticeship, the length of the medical curriculum and the manner of spending it are once more revived, not so much on their merits, but because England is engaged in a European war! Because of this we must again put the methods of medical education into the melting-pot and return to a system which had been abandoned as unsatisfactory. This is the conviction forced upon the mind of a gentleman who even before the war was satisfied that some such change was advisable. Now that war is upon us he is more certain than ever.

On another side is a writer who has been driven by the war to an almost passionate declaration of affection for "our excellent British system of medical education." He has long been both a teacher and an examiner under this system, and at the moment his pride in it is brightened with the glow of patriotism. How he manages to drag in the war as an agency helping to support what is evidently a favourite thesis is not a little curious. As readers of the daily Press know, steady efforts have been made in numerous and penetrating letters to the Editor to show that in all possible directions the Germans have really been very much overrated. It is here that the chance of the "excellent British system" comes in. In the first place, agreement is expressed with those who contest Germany's claim to be the herald of culture and who deny her possession of great names in literary, philosophic, and artistic activities. Nay, more; we are assured—it is a consulting surgeon who speaks—that an equal poverty marks the German achievements in "agriculture, irrigation, reclamation and drainage, polar and geographical exploration, sanitation, and various branches of surgery and medicine." After this general and comprehensive attack, a more detailed challenge is delivered on the claims made by Germany for excellence in the world of music, and these claims are examined with sternness and even with severity. If there are great names, the holders lived when peace and not war was the national ideal; or if they were Germans they were certainly not Prussians; or if they were born in Prussian towns the event took place before the Prussians arrived; or while one was indeed a Berliner, he was only a Jew Berliner. This being set out, the general conclusion is advanced that the pretensions of Teutonic professors ought not to be taken at their face value, and that we ought not to be content to sit obediently at their feet. Now comes the application. The last Royal Commission on the University of London proposed certain considerable changes in the conduct of medical education in the Metropolis. These changes, our surgeon tells us, would "Germanise" the University, and naturally the British patriot ought to refuse with scorn a proposal to regulate our academic affairs on lines parallel to those selected by a people whose single great musical achievement is the production of a "Jew Berliner." Perish the thought, and live for ever "our excellent British system"! At first sight one would have said that the war would suspend such domestic controversies as those concerned with the medical curriculum and the University of London. But experience proves the contrary, and shows how a national emergency may provide new arguments for combatants already equipped with secure convictions and ever ready for the fray.

